# Implementation of the American Society of Anesthesiologists 2022 paediatric guidelines in a child with mandibular metastasis

**DOI:** 10.1002/anr3.12274

**Published:** 2024-01-06

**Authors:** M. Larkins, J. Iasiello, K. Travia, M. Pasli, S. Cai, A. Hutton

**Affiliations:** ^1^ East Carolina University Brody School of Medicine North Carolina USA; ^2^ East Carolina Anesthesia Associates North Carolina USA

**Keywords:** ASA 2022 Practice Guidelines, Managing Difficult Airways, Paediatric Airway

## Abstract

The 2022 American Society of Anesthesiologists Practice Guidelines for Management of the Difficult Airway differ significantly from prior guidelines, particularly regarding paediatric patients. These guidelines place new emphasis on establishing a multidisciplinary team led by an anaesthetist trained in paediatric anaesthesia. Here, we demonstrate the clinical application of the new guidelines by presenting the case of a 16‐month‐old girl with a rapidly growing mandibular mass. The new guidelines stipulated the need for multidisciplinary team assembly; planning with indirect laryngoscopy; the availability of surgical tracheostomy and extracorporeal membrane oxygenation; and multiple ‘time out’ stops to confirm team members and plans. The patient tolerated induction of general anaesthesia and mask‐ventilation and tracheal intubation was achieved uneventfully on the first attempt. Her trachea was extubated uneventfully 5 days later. We emphasise the importance of paediatric anaesthesia training and videolaryngoscopy and discuss components of the 2022 American Society of Anesthesiologists Practice Guidelines for Management of the Difficult Airway with reference to a successful outcome in a paediatric difficult airway scenario.

## Introduction

The 2022 American Society of Anesthesiologists (ASA) Practice Guidelines for Management of the Difficult Airway (the 2022 ASA Guidelines) represent a significant change from previous airway guidelines, especially regarding paediatric patients [1]. The first ASA Practice Guidelines for Management of the Difficult Airway were published in 1993, in part to reduce adverse events such as airway trauma, brain or cardiac injury, and death [2]. The 2022 ASA Guidelines represent a revision of the 2013 ASA Guidelines [3]. Key points in the new guidelines include emphasis on a time‐out before procedure execution; minimising the number of attempts at tracheal intubation by emphasising airway management by those with the highest level of training and the use of videolaryngoscopy; and early consideration of extracorporeal membrane oxygenation (ECMO) for airway management failure [4]. Finally, the 2022 ASA Guidelines propose an algorithm for children with difficult airways, starting with a time‐out.

Neuroblastoma, an embryonal neoplasm of neural crest cell origin, is the most common extracranial tumour in children and is responsible for nearly 15% of all paediatric cancer deaths [5]. Metastasis to the mandible is rare; only 1% of all tumours in this region represent metastatic disease [6]. Metastatic disease of the mandible often presents with rapid mandibular swelling and such lesions, as with any pathology of the oral cavity, have the potential to cause airway compromise if not addressed quickly and appropriately [7]. This case demonstrates the utility of the 2022 ASA Guidelines in difficult paediatric patients.

## Report

A 16‐month‐old girl with no significant past medical history presented to a rural tertiary care centre for a scheduled meta‐iodobenzylguanidine scan and bone marrow biopsy for a rapidly growing right mandibular mass. Her family had noted decreased feeding in the past 2 weeks with almost complete cessation of oral intake in the past 2 days. The patient had no history of previous tracheal intubation or exposure to general anaesthesia.

**Figure 1:**
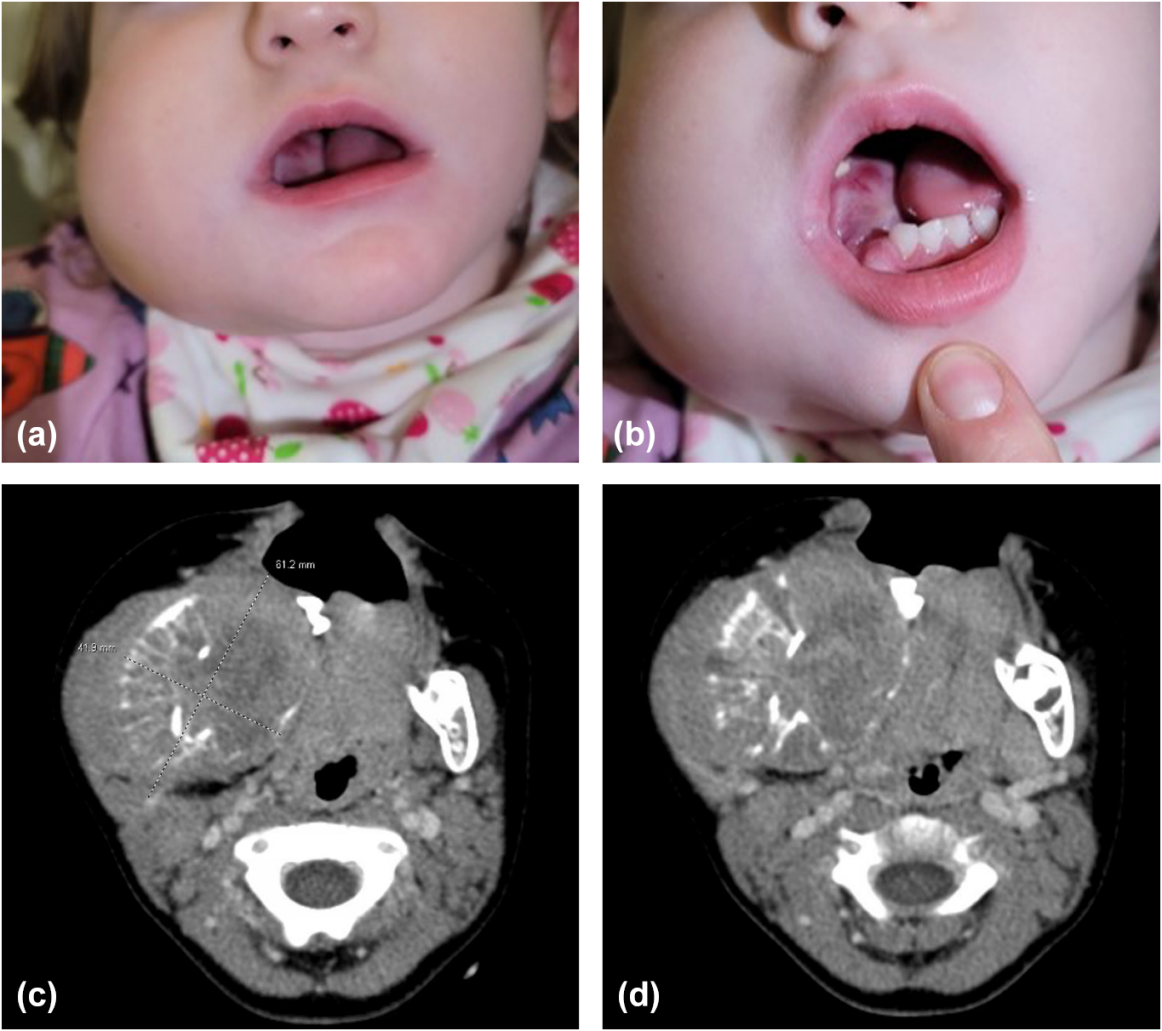
(a, b) Pre‐intubation photos of the patient's right mandibular mass, later diagnosed as metastatic neuroblastoma; (c) computed tomography (CT) scan of the patient's head and neck, where the right mandibular metastasis measures 6.1 cm at the widest diameter in the jaw; (d) CT showing midline shift but no local invasion of the patient's epiglottis.

Initial assessment and physical examination by the anaesthesia team revealed a firm, dense mass in the right oral cavity obstructing approximately 60% of the intra‐oral space (Fig. 1a and b). Significant secretions, tooth displacement and leftward tongue deviation were noted. Recognising a potentially difficult airway, the paediatric recommendations from the ASA 2022 Guidelines were reviewed and implemented. A multidisciplinary team was assembled consisting of a paediatric oncologist, an otolaryngologist, a paediatric surgeon, an extracorporeal membrane oxygenation (ECMO) specialist (perfusionist) and a paediatric anaesthetist. Computed tomography (CT) imaging of the patient's head and neck revealed a 6.1 cm tumour within the right mandibular region (Fig. 1c). Mass effect was seen, causing effacement of the oropharynx, hypopharynx and larynx with slight epiglottic deviation to the right (Fig. 1d). There was no tracheal deviation. No definite invasion of these structures was noted.

We opted to use videolaryngoscopy for the first attempt at tracheal intucation, with the most experienced paediatric anaesthetist present to minimise the number of attempts. We considered oral versus nasal intubation, as CT imaging indicated an unobstructed nasopharynx. A fibreoptic bronchoscope was prepared and within reach. Elective tracheostomy was also considered and relegated to a backup option in addition to ECMO, to be utilised upon failure to secure an airway.

Time‐out was performed with all team members present and there was confirmation of each team's plans, including induction of general anaesthesia, primary and backup airway management options and surgical approach. Inhalational anaesthetic induction with sevoflurane and gentle bag‐mask ventilation confirmed the ability to ventilate the patient's lungs. Following administration of rocuronium 5 mg and ketamine 40 mg, tracheal intubation was achieved on the first attempt with a C‐MAC D‐blade (Karl Storz SE & Co, Tuttlingen, Germany) and 4.0 mm tracheal tube. The laryngeal view was deemed a Fremantle Score of P (partial glottic view) 1 (easy tracheal intubation) C‐MAC (device) [8].

Tracheal intubation was maintained for 5 days to facilitate multiple procedures and allow time for chemotherapy to shrink the mass. Subsequent imaging indicated that the patient had an adrenal mass consistent with neuroblastoma and a biopsy confirmed that the mandibular mass was a metastatic lesion. The management of tracheal extubation was in accordance with the ASA 2022 Guidelines: the patient was placed on 100% oxygen and her trachea was extubated followed by application of supplemental oxygen via a nasal cannula. A videolaryngoscope, laryngeal mask airway and emergency cricothyrotomy catheter set were all assembled at bedside before extubation. A brief arterial oxygen desaturation to 75% occurred after tracheal extubation, which resolved spontaneously to 98%. The remainder of the patient's hospital course was uneventful.

## Discussion

Previous guidelines, such as the 2013 ASA Guidelines, make little mention of airway management in children [3]. Paediatric airways can pose an increased challenge due to variable anatomy, intolerance to apnoea and susceptibility to airway oedema, which has potentially more serious outcomes given the calibre of paediatric airways [9]. The main consideration in previous guidelines was the potential for paediatric patients to be uncooperative during care, but no algorithm or management guideline had been put forward beyond suggesting preoxygenation before intubation. There was no discussion of pre‐oxygenation or the use of supplemental oxygen for any patient around the time of tracheal extubation in the 2013 ASA Guidelines. The challenges associated with paediatric airways have driven interest and research into new methods to safely manage these patients, with proposed solutions focusing on improved teamwork and communication methods, increased use of videolaryngoscopy, and the creation of algorithms for stratifying and treating paediatric patients [10]. The 2022 ASA Guidelines also represent an international effort to standardise airway management and were created with input from various groups that manage airway standards across the world including the European Airway Management Society and the European Society of Anaesthesiology and Intensive Care.

In our case, given the rapidly growing mass and the potential for invasion of airway structures, early identification of a difficult airway was critical for safe tracheal intubation. It is important to note that the anaesthetist is identified as the driver for early identification, investigation and team leadership concerning difficult airways in the new 2022 ASA Guidelines [1], a role established since the Guidelines were first created in 1993 [2]. The patient originally planned to have this procedure performed on an outpatient basis; however, at outpatient assessment the mass was deemed to be a risk factor for difficult intubation, resulting in the paediatric anesthesiology team at our institution being consulted and the admission being switched to inpatient. It was only after the anaesthetist gathered a multidisciplinary team and discussed the plan for airway management that additional imaging and workup were performed, allowing for definitive information regarding the extent of the patient's metastatic disease. Had the Guidelines not been considered during intubation, potential epiglottic invasion may not have been considered. Based on the patient's imaging, showing a well‐circumcised mass without epiglottic or laryngeal invasion, tracheal intubation could be attempted initially, rather than planning for elective tracheostomy. Finally, having a perfusionist and a primed ECMO circuit on standby provided safety and comfort to the treatment team and family. A paediatric surgeon was available to secure the lines necessary for ECMO support.

The 2022 ASA Guidelines also advocate the use of videolaryngoscopy [1]. This is associated with improved success as it allows for better visualisation of the larynx, although it requires appropriately sized blades to be effective in children. Meta‐analyses reported in the 2022 ASA Guidelines cite increased frequency of successful first attempt and overall intubations using videolaryngoscopy, in addition to the need for fewer intubation manoeuvres [1]. Observational studies have demonstrated intubation success rates with videolaryngoscopy from 85 to 100% and first attempt success rates ranging from 51 to 100% [1]. Of note, videolaryngoscopy with paediatric blades is easily accessible at our institution. Other institutions, especially those in rural communities or other low‐income environments, may not have access to these technologies. Additionally, these institutions may lack access to providers experienced in securing paediatric airways. The widespread adoption of the 2022 ASA Guidelines may be limited in this regard, as access to both paediatric videolaryngoscopy and providers with the knowledge to appropriately use this equipment may place financial strain on disadvantaged institutions. This financial strain has been reported in the literature and was especially relevant during the COVID‐19 pandemic, where the overwhelming numbers of patients requiring hospitalisation and videolaryngoscopy for tracheal intubation prompted research into alternatives, such as the three‐dimensional printing of laryngoscopes [11]. As a final aside, the multidisciplinary team debrief following the patient's procedures identified the utility of having a videolaryngoscope equipped for paediatric patients and the value of having all team members (multi‐speciality surgeons, perfusionist, oncologist and anaesthetist) present for time‐out and contingency planning.

Finally, the 2022 ASA Guidelines also discuss tracheal extubation of patients with challenging airways with similar emphasis on features discussed in their intubation section [1]. A focus on having a preformulated strategy with appropriate equipment and staff nearby is discussed in the Guidelines. The consideration of an elective surgical tracheostomy and the use of supplemental oxygen are also discussed in the 2022 ASA Guidelines and were considered in our management of this case.

The 2022 ASA Guidelines emphasise multidisciplinary teamwork and the use of advanced airway techniques such as videolaryngoscopy, which proved successful in this case. The utility of these guidelines has yet to be reported in the literature and their widespread adoption is not without barriers, such as the financial strain associated with their implementation.
